# A Machine Learning-Based Model to Predict Survival After Transarterial Chemoembolization for BCLC Stage B Hepatocellular Carcinoma

**DOI:** 10.3389/fonc.2021.608260

**Published:** 2021-03-02

**Authors:** Huapeng Lin, Lingfeng Zeng, Jing Yang, Wei Hu, Ying Zhu

**Affiliations:** ^1^ Department of Intensive Care Unit, Affiliated Hangzhou First People’s Hospital, Zhejiang University School of Medicine, Hangzhou, China; ^2^ Department of Hepatobiliary Surgery, The Second Affiliated Hospital of Chongqing Medical University, Chongqing, China; ^3^ Department of Nephrology, The Second Xiangya Hospital of Central South University, Changsha, China

**Keywords:** hepatocellular carcinoma, BCLC Stage B, machine learning, random survival forest, prognosis

## Abstract

**Objective:**

We sought to develop and validate a novel prognostic model for predicting survival of patients with Barcelona Clinic Liver Cancer Stages (BCLC) stage B hepatocellular carcinoma (HCC) using a machine learning approach based on random survival forests (RSF).

**Methods:**

We retrospectively analyzed overall survival rates of patients with BCLC stage B HCC using a training (n = 602), internal validation (n = 301), and external validation (n = 343) groups. We extracted twenty-one clinical and biochemical parameters with established strategies for preprocessing, then adopted the RSF classifier for variable selection and model development. We evaluated model performance using the concordance index (c-index) and area under the receiver operator characteristic curves (AUROC).

**Results:**

RSF revealed that five parameters, namely size of the tumor, BCLC-B sub-classification, AFP level, ALB level, and number of lesions, were strong predictors of survival. These were thereafter used for model development. The established model had a c-index of 0.69, whereas AUROC for predicting survival outcomes of the first three years reached 0.72, 0.71, and 0.73, respectively. Additionally, the model had better performance relative to other eight Cox proportional-hazards models, and excellent performance in the subgroup of BCLC-B sub-classification B I and B II stages.

**Conclusion:**

The RSF-based model, established herein, can effectively predict survival of patients with BCLC stage B HCC, with better performance than previous Cox proportional hazards models.

## Introduction

Hepatocellular carcinoma (HCC) is the second leading cause of cancer-related deaths in the world (1–3). Its prognosis remains poor, owing to a relatively high proportion of unresectable disease at the time of diagnosis, although the Barcelona Clinic Liver Cancer (BCLC) staging system, endorsed by the European Association for the Study of the Liver (EASL) and the American Association for the Study of Liver Diseases (AASLD), have been extensively used in clinical practice (4). Patients with stage B BCLC are considered unsuitable for curative treatment, and their overall survival rates are varied mainly due to heterogeneity of liver function and tumor burdens (5). Consequently, several subclassification systems or risk predication models for BCLC stage B HCC patients have been proposed.

The subclassification system proposed in 2012 categorized patients with intermediate HCC into four substages, namely B1 to B4 (6). The following year, Kadalayil et al. developed a simple prognostic score which is entitled HAP score with several parameters including albumin, bilirubin, *α*-fetoprotein (AFP), and tumor size (7). Recently, an inflammation biomarker was shown to be a prognostic predictor for cancer patients, whereas Chon developed and validated a nomogram, including neutrophil-to-lymphocyte ratio, for predicting survival rates of patients with intermediate HCC (8). Despite these advancements, all aforementioned models were based on the traditional Cox proportional-hazards approach.

Although several prognostic models have been established, no tool exists that can effectively estimate survival outcomes after TACE for BCLC stage B HCC. Previous studies have reported the potential for integrated machine learning algorithms in developing effective models to predict risk factors associated with survival outcomes (9). Particularly, this approach enhances understanding of patterns and hidden relationships between factors that could be missed when traditional biostatistical methods are used (10, 11). Among known machine-learning classifiers, the random forest classifier offers excellent performance in modeling and has subsequently been used in management of right-censored survival data. The resulting RSF is a non-parametric classifier that provides variable importance values for all candidate predictors (12). In the present work, we evaluated whether RSF could predict survival outcomes of patients with BCLC stage B HCC. Additionally, we assessed the importance and predictive value of clinical variables for prognostic outcome and compared RSF-derived results with those previously obtained using Cox proportional-hazards models.

## Methods

### Study Population and Selection Criteria

We retrospectively recruited 979 consecutive patients with BCLC Stage B HCC from a database (13), between January 2007 and December 2016. The inclusion criteria were: (1) adult patients diagnosed with HCC according to the AASLD guidelines; (2) patients with liver function of Child–Pugh class A or B; (3) patients with an Eastern Cooperative Oncology Group (ECOG) performance status of 0; (4) patients with multiple tumors and no vascular invasion or lymphatic/extrahepatic metastasis; and (5) patients who had complete follow-up by magnetic resonance imaging or computed tomography and bio-chemical routine test. The exclusion criteria were: (1) patients with a history of malignancies other than HCC; (2) those who manifested recurrent HCC or HCC with vascular invasion or lymphatic/extrahepatic metastasis; (3) patients with a liver function of Child–Pugh class C; (4) those with hepatic encephalopathy/refractory ascites/gastrointestinal hemorrhage; (5) patients with immunodeficiency or autoimmune disease; and (6) those whose follow-up duration was less than three months. All patients were divided into training and validation groups, at a ratio of two to one, then an individual cohort comprising 414 patients from the same database was used for external validation. All patients in the external validation cohorts came from different hospitals from the primary cohort.

### Establishment of the Prognostic Model

We collected demographic and biochemical parameters from all patients for analysis. These included their age, gender, virus infection status, hemoglobin level, white blood cell count, platelet count (PLT), aspartate aminotransferase (AST), albumin, total bilirubin, c-reactive protein (CRP), prothrombin time (PT), ascites, alpha-fetoprotein, tumor number and size, tumor vascular invasion, distant or lymph node metastasis, and performance status score. We evaluated the Child–Pugh grade using laboratory data from albumin, PT, and total bilirubin, as well as clinical data of hepatic encephalopathy and ascites. Particularly, the ascites were defined as the radiological ascites, whereas the AST to platelet ratio index (APRI) was calculated using the following formula: ([AST/upper limit of normal]/platelet count [10^9^)/L]) × 100. On the other hand, the ALBI score was calculated as follows: linear predictor = (log10 bilirubin x 0.66) + (albumin × −0.085), where bilirubin is in mol/L and albumin in g/L. Additionally, the BCLC-B sub-classification was as previously described by Bolondi L (6). Overall survival comprised primary outcomes and was defined as the time from HCC diagnosis to last follow-up. Patients were followed up monthly, during the period of initial treatment, then after every 2 to 3 months for the first 2 years if complete remission was achieved. Frequency of follow-up gradually decreased to every 3 to 6 months after 2 years’ remission. Overall survival rates were estimated using the Kaplan–Meier method, with the log-rank test used to compare survival curves.

Thereafter, we selected prognostic factors based on the RSF classifier method, with permutation-based selection conducted using the variable importance (VIMP) metric of the RSF. For VIMP, a random subset of predictor variable values was permuted then the difference in prediction error, between the observed and randomly permutated variables, calculated as previously described (14, 15). Summarily, a high VIMP suggests that misspecification worsens predictive accuracy in the forest, whereas a low VIMP suggests that noise is more informative than the observed variable. The resulting top five risk factors, with the highest VIMP, are chosen for model development by the RSF classifier. We validated the selected variables using the minimal depth and the frequency form the 10-fold cross validation.

### Statistical Analysis

Continuous variables were presented as means with standard deviation (SD) of the means or median with interquartile ranges (IQR), whereas categorical ones were presented as percentages. We adopted the multiple imputation method for missing data, and trained RSF by growing a large number of individual trees with each tree trained on a random-bootstrap sample from the original cohort, followed by a 10-fold cross validation. Starting with the entire sample at the tree trunk, we chose a random set of variables as candidates for splitting the branch into two subbranches, with the aim of maximizing the difference in survival between subbranches. We determined optimal splitting threshold for each candidate variable, then chose the variable with maximum log-rank statistic between split data for splitting. This process was repeated until a predetermined terminal node size was achieved. A trained random survival forest predicts an individual mortality, which was calibrated on the number of events. Specifically, if all patients shared similar characteristics, the predicted mortality would be equal to the number of expected deaths. To evaluate the predictive performance of the random survival forest, we calculated concordance index (c-index) of the final forest, then evaluated accuracy of the predicted outcome using AUROC. Additionally, we compared our model’s performance with previously established ones, such as the HAP score, the mHAP II score, the ALBI-TAE model, as well as the up-to-seven, four-and-seven, six-and-twelve score, BCLC-B sub-staging and the New BCLC B sub-staging systems. All statistical analyses were performed using packages implemented in R software (version 3.5), with statistical significance set at p<0.05.

## Results

### *Patient* Characteristics

**Figure 1:**
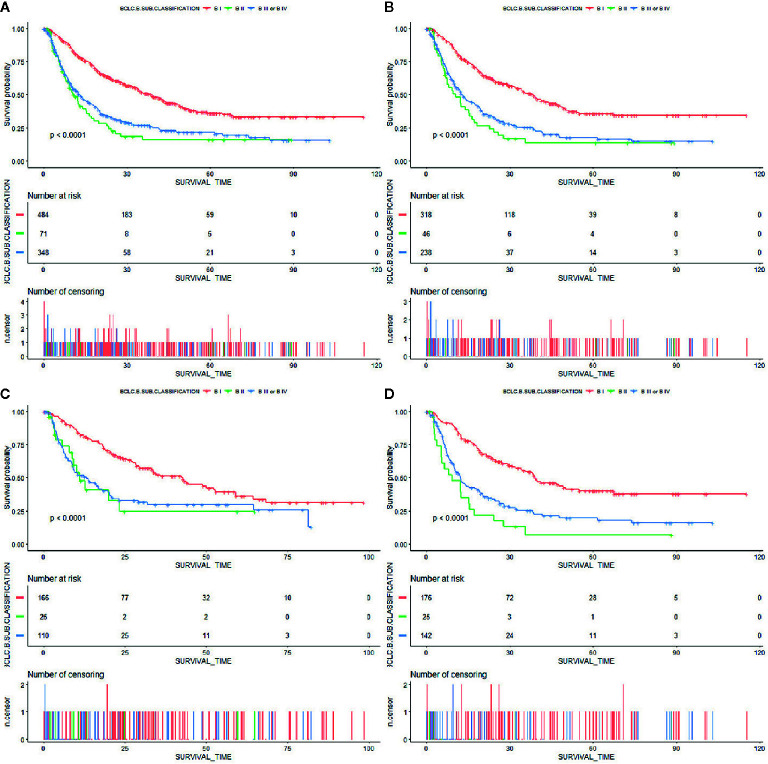
Kaplan–Meier curves of overall survival in patients with BCLC stage B HCC stratified by BCLC-B sub-classification in the **(A)** primary cohort, **(B)** training cohort, **(C)** internal validation cohort, and **(D)** external validation cohort.

A total of 903, out of 979, patients met the inclusion criteria and were therefore used for model development and validation. 602 and 301 patients were placed into training and internal validation cohorts, respectively. Their baseline characteristics are presented in **Table 1**. Summarily, median follow-up periods for the training and validation cohorts were 17.6 and 17.0 months, respectively. Most of the patients were infected with HBV, with only a handful infected with HCV. This may be because the included patients were all from Asia. Almost all clinical parameters, except Child–Pugh and ALBI grades, were well-balanced between the training and validation groups. The percentage of patients of Child–Pugh A in the training group was more than that in the validation group, with more ALBI grade I patients found in the validation than in the training group. A total of 343 patients were used for external validation. Their baseline characteristics are summarized in **Supplementary Table 1**. Patients in BCLC-B sub-classification B I stage had a significantly better overall survival than the others (**Figure 1**). However, the Child–Pugh score could hardly distinguish patients with diverse prognosis (**Supplementary Figure 1**).

**Table 1:** The baseline characteristics of the BCLC stage B HCC patients.

The Variables	The Patients (n = 903)		P value
	Training group (n = 602)	Validation group (n = 301)	
**Gender, n (%)**			0.672
**Male**	401 (66.6%)	219 (72.8%)	
**Female**	201 (33.4%)	82 (27.2%)	
**Age (years), mean (SD)**	52.8 (12.5)	53.8 (11.9)	0.246
**HBsAg, n (%)**			0.357
**Negative**	73 (3.1%)	38 (4.0%)	
**Positive**	529 (96.9%)	263 (96.0%)	
**Anti-HCV, n (%)**			0.134
**Negative**	531 (97.3%)	262 (95.6%)	
**Positive**	15 (2.7%)	12 (4.4%)	
**HGB (g/L), mean (SD)**	132.6 (19.8)	132.2 (19.5)	0.749
**WBC (10^9^/L), median (IQR)**	7.1 (5.4–9.4)	6.5 (4.8–9.1)	0.407
**LDH (U/L), median (IQR)**	228.8 (185.4–340.0)	235.3 (185.3–385.5)	0.758
**PLT (10^9^/L), median (IQR)**	142.0 (98.0–201.0)	148.4 (100.8–206.0)	0.367
**AST (U/L), median (IQR)**	68.6 (40.9–132.7)	60.4 (37.0–108.4)	0.498
**ALB (g/L), mean (SD)**	38.6 (5.8)	38.9 (5.5)	0.531
**TBLT (umol/L), median (IQR)**	18.8 (12.8–27.0)	17.4 (12.3–27.5)	0.396
**CRP (mg/L), median (IQR)**	14.2 (3.3–51.8)	19.9 (3.6–63.8)	0.082
**PT (seconds), mean (SD)**	12.3 (1.3)	12.3 (1.4)	0.896
**AFP (ng/ml), median (IQR)**	260.0 (15.6–4427.8)	204.1 (21.3–4456.0)	0.941
**Size of main tumor (mm), median (IQR)**	64.5 (42.0-93.0)	66.0 (44.0-99.0)	0.402
**Number of lesions, n (%)**			0.222
**≤2**	183 (30.4%)	101 (33.6%)	
**>2**	419 (69.6%)	200 (66.4)	
**Ascites, n (%)**			0.279
**No**	575 (95.5%)	292 (97.0%)	
**Yes**	27 (4.5%)	9 (3.0%)	
**Child-Pugh score, n (%)**			0.008
**5**–**6**	86 (14.3%)	64 (21.3%)	
**7**–**9**	516 (85.7%)	237 (78.7%)	
**BCLC-B sub-classification**			0.015
**B I**	362 (60.1%)	122 (40.5%)	
**B II**	51 (8.5%)	20 (6.6%)	
**B III or B IV**	165 (27.4%)	183 (60.8%)	
**ALBI grade**			0.029
**I**	460 (76.4%)	249 (82.7%)	
**II**	142 (23.6%)	52 (17.3%)	
**APRI score, median (IQR)**	1.3 (0.7–2.9)	1.0 (0.6–2.3)	0.876

HCC, hepatocellular carcinoma; HCV, hepatitis C virus; HGB, hemoglobin; WBC, white blood cell; LDH, lactate dehydrogenase; PLT, platelet; AST, aspartate aminotransferase; ALB, albumin; TBLT, total bilirubin; CRP, c-reactive protein; PT, prothrombin time; AFP, alpha-fetoprotein; ALBI, albumin-bilirubin grade; APRI, AST to Platelet Ratio Index; SD, standard deviation; IQR, interquartile range.

### RSF Models

**Figure 2:**
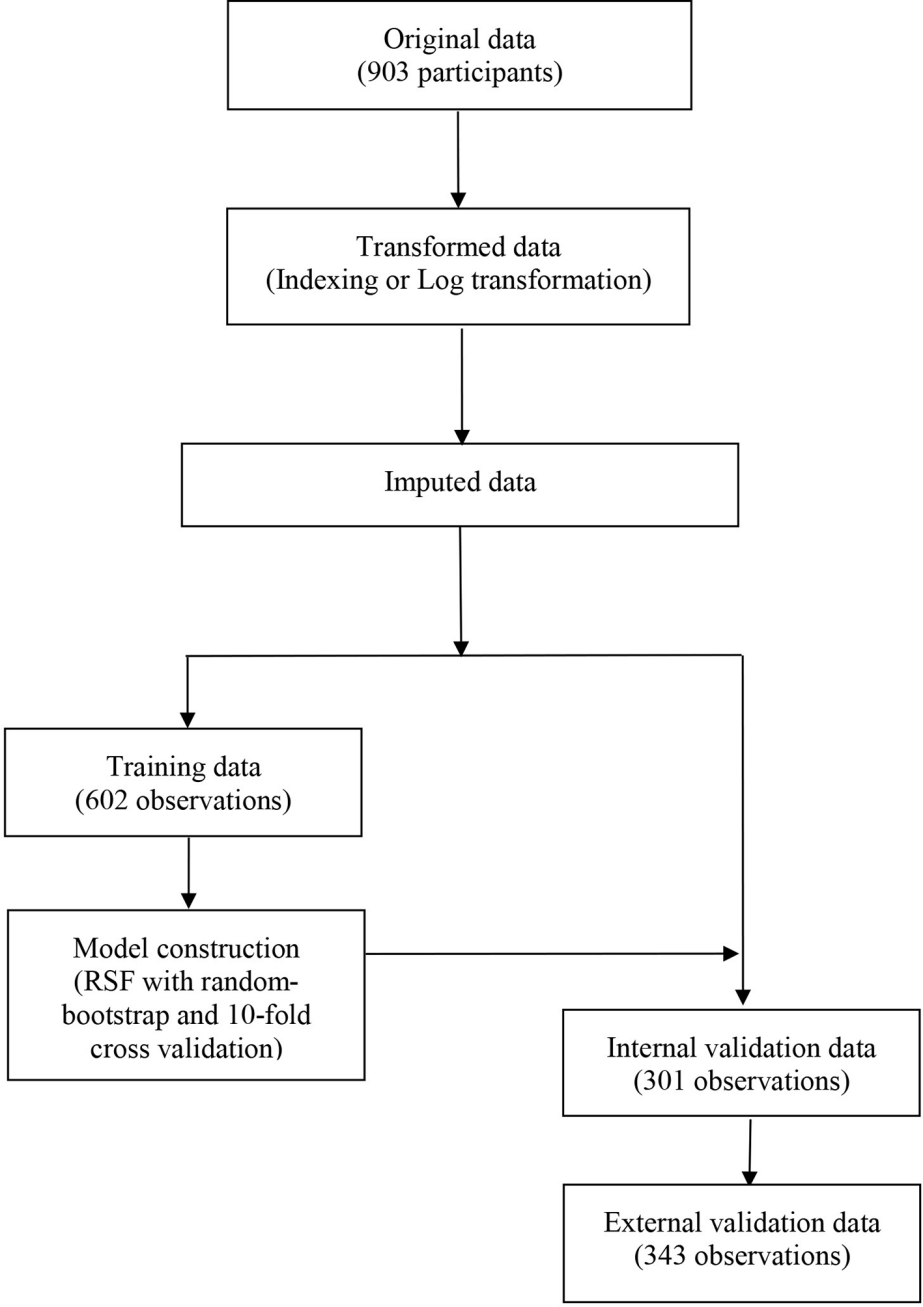
The flowchart describing the general framework of the study. Models were built using the training dataset and validated in the internal and external validation cohorts.

**Figure 3:**
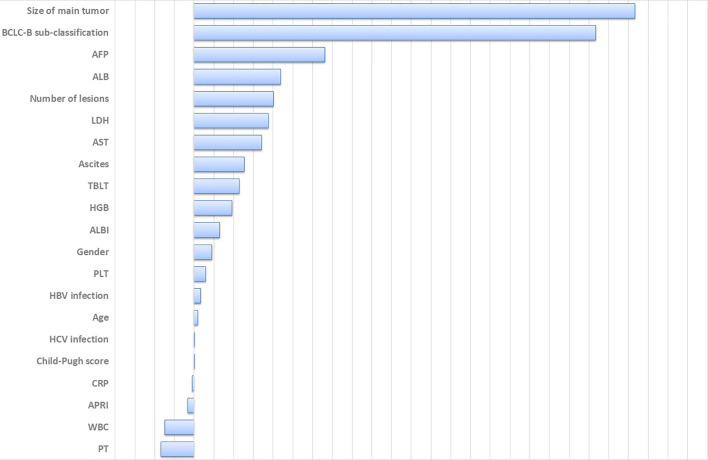
Variable importance of all clinical parameters. Large positive values indicate predictive variables, whereas zero or negative importance values identify no predictive variables. HCV, hepatitis C virus; HGB, hemoglobin; WBC, white blood cell; LDH, lactate dehydrogenase; PLT, platelet; AST, aspartate aminotransferase; ALB, albumin; TBLT, total bilirubin; CRP, c-reactive protein; PT, prothrombin time; AFP, alpha-fetoprotein; ALBI, albumin-bilirubin grade; APRI, AST to Platelet Ratio Index.

**Figure 4:**
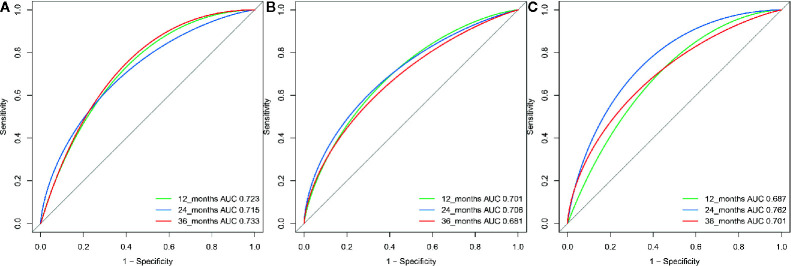
The ROC curve for the RSF-based model for predicting survival at year 1, year 2, and year 3 in the **(A)** training, **(B)** internal validation group and **(C)** external validation group.

A total of 21 covariates, including clinical variables and laboratory data, were collected at baseline and were considered candidates for analysis and modeling. All statistical analysis procedures used in this study are outlined in **Figure 2**. Data transformation, indexing, and imputation were performed to generate data points for predicting overall survival rates during the follow-up period. Summarily, all variables were ranked according to the VIMP after the RSF (**Figure 3**). A detailed description of the VIMP and minimal depth of each variable are listed in **Supplementary Table 2**. Briefly, a total of 17 and four variables had positive and negative values, respectively. In addition, tumor size, BCLC-B sub-classification, AFP and ALB levels, as well as number of lesions exhibited the highest VIMP and lowest minimal depth, indicative of strong predictive performance. Consequently, these five parameters were used to establish the RSF model. The trained random survival forest achieved a concordance index of 0.69 (0.66–0.71), with the AUROC for predicting survival outcomes in the first three years reaching 0.72, 0.71, and 0.73 respectively (**Figure 4A**).

### *Model* Validation *and* Comparison

We validated model performance using the validation group. Specifically, AUROC-based prediction of survival outcomes for the first three years reached 0.70, 0.71, and 0.68 respectively, in the internal validation cohorts, whereas that in the external validation cohort reached a respective 0.69, 0.76, and 0.70 (**Figures 4B, C**). A comparison between our model with eight others (6, 7, 16–21), including the HAP and mHAP II scores, the ALBI-TAE model, as well as the up-to-seven, the four-and-seven, the six-and-twelve score, the BCLC-B sub-staging, and the New BCLC B sub-staging systems, indicated that ours had the highest c-index (**Table 2**).

**Table 2:** The comparison of the present model versus other models for BCLC stage B HCC. patients.

Model	1-yr AUROC	2-yr AUROC	3-yr AUROC	C-index (95% CI)
**Training group**				
**The present model**	0.72	0.71	0.73	0.69 (0.66–0.71)
**Up-to-seven**	0.62	0.63	0.62	0.59 (0.57–0.61)
**Four-and-seven**	0.65	0.63	0.62	0.62 (0.60–0.64)
**Six-and-twelve**	0.66	0.64	0.62	0.63 (0.61–0.65)
**BCLC-B sub-staging system**	0.60	0.59	0.59	0.59 (0.57–0.61)
**New BCLC B sub-staging system**	0.61	0.60	0.59	0.59 (0.57–0.62)
**HAP**	0.60	0.59	0.58	0.58 (0.56–0.61)
**mHAP II**	0.56	0.56	0.55	0.55 (0.53–0.57)
**ALBI-TAE model**	0.67	0.65	0.64	0.64 (0.62–0.67)
**Internal validation group**				
**The present model**	0.70	0.71	0.68	0.70 (0.67–0.74)
**Up-to-seven**	0.62	0.61	0.60	0.61 (0.59–0.63)
**Four-and-seven**	0.65	0.63	0.61	0.63 (0.59–0.66)
**Six-and-twelve**	0.64	0.64	0.63	0.63 (0.60–0.66)
**BCLC-B sub-staging system**	0.64	0.63	0.62	0.63 (0.59–0.66)
**New BCLC B sub-staging system**	0.65	0.63	0.62	0.64 (0.60–0.67)
**HAP**	0.61	0.61	0.59	0.60 (0.56–0.63)
**mHAP II**	0.57	0.58	0.56	0.57 (0.54–0.60)
**ALBI-TAE model**	0.69	0.67	0.65	0.67 (0.63–0.70)
**External validation group**				
**The present model**	0.69	0.76	0.70	0.69 (0.64–0.73)
**Up-to-seven**	0.61	0.60	0.59	0.59 (0.56–0.61)
**Four-and-seven**	0.68	0.66	0.65	0.65 (0.62–0.68)
**Six-and-twelve**	0.67	0.65	0.63	0.63 (0.60–0.66)
**BCLC-B sub-staging system**	0.63	0.61	0.60	0.61 (0.57–0.63)
**New BCLC B sub-staging system**	0.66	0.64	0.63	0.63 (0.59–0.66)
**HAP**	0.60	0.59	0.59	0.59 (0.54–0.62)
**mHAP II**	0.57	0.57	0.56	0.56 (0.23–0.59)
**ALBI-TAE model**	0.67	0.66	0.65	0.64 (0.60–0.68)

AUROC, area under the receiver operating characteristics curves; CI, confidence interval.

### Individual Analysis of BI and BII Stages

The use of TACE in BCLC-B sub-classification B I and B II patients is a controversial topic, with liver transplantation deemed an alternative choice for this group of patients. Patients with B I stage had significantly better overall survival rates relative to their B II stage counterparts. Consequently, we performed an individual analysis for B I and B II patients and found that the present model worked well in both groups of patients after TACE. Specifically, the AUROC for predicting survival outcomes in the 1^st^, 2^nd^ and 3^rd^ years reached 0.78, 0.76, and 0.73, respectively in the training, a respective 0.76, 0.73, and 0.74 in the internal validation, and a respective 0.72, 0.71, and 0.69 in the external validation cohorts. Additionally, our model had excellent performance in the subgroup of B I patients. Overall, this model has potential for selecting patients unsuitable for TACE-based treatment in B I and B II stage subgroups.

## Discussion

In the present study, we used RSF, a machine learning-based algorithm, to establish a model for predicting survival outcomes of patients with BCLC stage B HCC. Based on VIMP, we identified and evaluated five parameters, namely tumor size, BCLC-B sub-classification, AFP, and ALB levels, as well as number of lesions as strong predictors. These were subsequently used for establishment of the model. A comparison between our and other traditional Cox proportional-hazards models revealed that the present model is an effective tool for estimating survival outcomes after TACE for patients with BCLC stage B HCC.

Previously developed predictive models for patients with intermediate HCC are all based on the traditional Cox proportional-hazards method, which is limited by the possibility of over-fitting, data mining purposes due to correlation between variables, or non-linearity of variables (including potential complex interactions among them) (4, 22). Recently, a machine-learning based statistical model, called RSF has emerged as an intuitive technique for predicting individual risk in cancer patients. This method has potential for establishing predictive models, especially in cases where response variables are censored survival data and the relationship between response and predictor is complex. In fact, recent studies have proved its efficacy in treatment responses and predicting survival outcome events in several types of cancer (14).

Based on bootstrap data and numerous lines of evidence from individual decision trees, it is evident that RSF offers the following advantages: 1) it allows for an intuitive assessment of variable importance; 2) it can deal with correlated parameters, variable interactions, and non-linear effects; and 3) it requires little input from the analyst. Additionally, RSF does not rely on restrictive assumptions, in contrast with traditional Cox proportional-hazards models (23). In the present study, our model revealed that several predictors, namely, tumor sizes, AFP level, and the number of lesions were strong predictors, consistent with previous studies. And ALB level was shown to be an effective tool for assessing liver function and has subsequently been adopted as a prognostic marker for HCC (23–25). Several traditional prognostic factors, such as ALBI, were not ranked high in the present model, possibly because those factors are fundamental to development, maintenance, and progression of HCC death. Additionally, they are intrinsic components of other risk factors, particularly sub-clinical ones that are more distal to disease initiation but closer to adverse outcomes.

This study had several limitations. Firstly, the inherent limitations associated with a retrospective study. Secondly, the AUROC was low and should be validated using other cohorts. Thirdly, all participants were from the Asian centers. These findings need to be validated using western populations. Fourthly, despite the included patients receiving TACE as a first-line treatment therapy, additional treatments, such as radioembolization, targeted therapy or ablation therapy, during the follow-up period may have influenced survival rates, although these need not be controlled. Fifthly, we only included 21 clinical parameters in our analysis, although other parameters such as genetics and imaging features could also be informative in the modeling. Lastly, the used database did not provide definitions for multiple lesions, while the data on how far apart the lesions were could be included in the future study.

In conclusion, we used RSF-based approach to successfully develop a model for predicting survival rates of patients with BCLC stage B HCC. This model guarantees superior performance compared to previously published Cox proportional hazards models.

## Data Availability Statement

The raw data supporting the conclusions of this article will be made available by the authors, without undue reservation.

## Author Contributions

All authors collected, extracted, and analyzed the data and wrote the article. HL and YZ conceived and designed this study. All authors contributed to the article and approved the submitted version.

## Conflict of Interest

The authors declare that the research was conducted in the absence of any commercial or financial relationships that could be construed as a potential conflict of interest.
