# Supplementary material for: A Machine Learning-Based Model to Predict Survival After Transarterial Chemoembolization for BCLC Stage B Hepatocellular Carcinoma
Source: Front Oncol. 2021 Mar 2;11:608260. doi: 10.3389/fonc.2021.608260 (PMC7962602; doi:10.3389/fonc.2021.608260)

**Supplementary Table 1.** The baseline characteristics of the BCLC stage B HCC patients from the external validation cohort.

| **The Variables** | **The External Validation Cohort (n=343)** |
| --- | --- |
| **Gender, n (%)** |  |
| **Male** | 286 (83.4%) |
| **Female** | 57 (16.6%) |
| **Age (years), mean (SD)** | 52.0 (11.9) |
| **AST (U/L), median (IQR)** | 63.0 (38.0-119.0) |
| **ALB (g/L), mean (SD)** | 38.7 (6.0) |
| **TBLT (umol/L), median (IQR)** | 19.4 (12.9-27.2) |
| **PT (seconds), mean (SD)** | 12.3 (1.2) |
| **AFP (ng/ml), median (IQR)** | 210.2 (12.0-1988.0) |
| **Size of main tumor (mm), median (IQR)** | 67.0 (43.0-96.5) |
| **Number of lesions, n (%)** |  |
| **≤3** | 124 (36.2%) |
| **>3** | 219 (63.8%) |
| **Ascites, n (%)** |  |
| **No** | 326 (95.0%) |
| **Yes** | 17 (5.0%) |
| **Child-Pugh grade, n (%)** |  |
| **A** | 298 (86.9%) |
| **B** | 45 (13.1%) |
| **Child-Pugh score, n (%)** |  |
| **≤6** | 298 (86.9%) |
| **7** | 29 (8.5%) |
| **8** | 16 (4.7%) |
| **≥9** | 0(0%) |
| **BCLC-B sub-classification** |  |
| **B I** | 273 (79.6%) |
| **B II** | 46 (13.4%) |
| **B III or B IV** | 24 (6.9%) |
| **ALBI score, mean (SD)** | -2.4 (0.6) |

**Supplementary Table 2.** The VIMP and minimal depth of the variables.

| **Variables** | **VIMP** | **Minimal depth** |
| --- | --- | --- |
| **Size of main tumor** | 0.0223 | 2.036 |
| **BCLC-B sub-classification** | 0.0203 | 3.143 |
| **AFP** | 0.0066 | 3.579 |
| **ALB** | 0.0044 | 4.153 |
| **Number of lesions** | 0.0040 | 4.546 |
| **LDH** | 0.0037 | 4.56 |
| **AST** | 0.0034 | 4.652 |
| **Ascites** | 0.0025 | 5.003 |
| **TBLT** | 0.0023 | 4.741 |
| **HGB** | 0.0019 | 5.696 |
| **ALBI** | 0.0013 | 5.600 |
| **Gender** | 0.0009 | 5.687 |
| **PLT** | 0.0005 | 5.239 |
| **HBV infection** | 0.0003 | 6.264 |
| **Age** | 0.0002 | 5.717 |
| **HCV infection** | 3.13E-05 | 5.160 |
| **Child-Pugh score** | 2.89 E-05 | 6.344 |
| **CRP** | -0.0001 | 4.850 |
| **APRI** | -0.0003 | 5.201 |
| **WBC** | -0.0016 | 6.696 |
| **PT** | -0.0017 | 6.571 |

Vimp, variable importance; HCV, hepatitis C virus; HGB, hemoglobin; WBC, white blood cell; LDH, lactate dehydrogenase; PLT, platelet; AST, aspartate aminotransferase; ALB, albumin; TBLT, total bilirubin; CRP, c-reactive protein; PT, prothrombin time; AFP, alpha-fetoprotein; ALBI, albumin-bilirubin grade; APRI, AST to Platelet Ratio Index; SD, standard deviation; IQR, interquartile range.

**Supplementary Figure 1. Kaplan-Meier curves of overall survival in patients with BCLC stage B HCC stratified by Child-Pugh score in the A) primary cohort, B) training cohort, C) internal validation cohort and D) external validation cohort.**


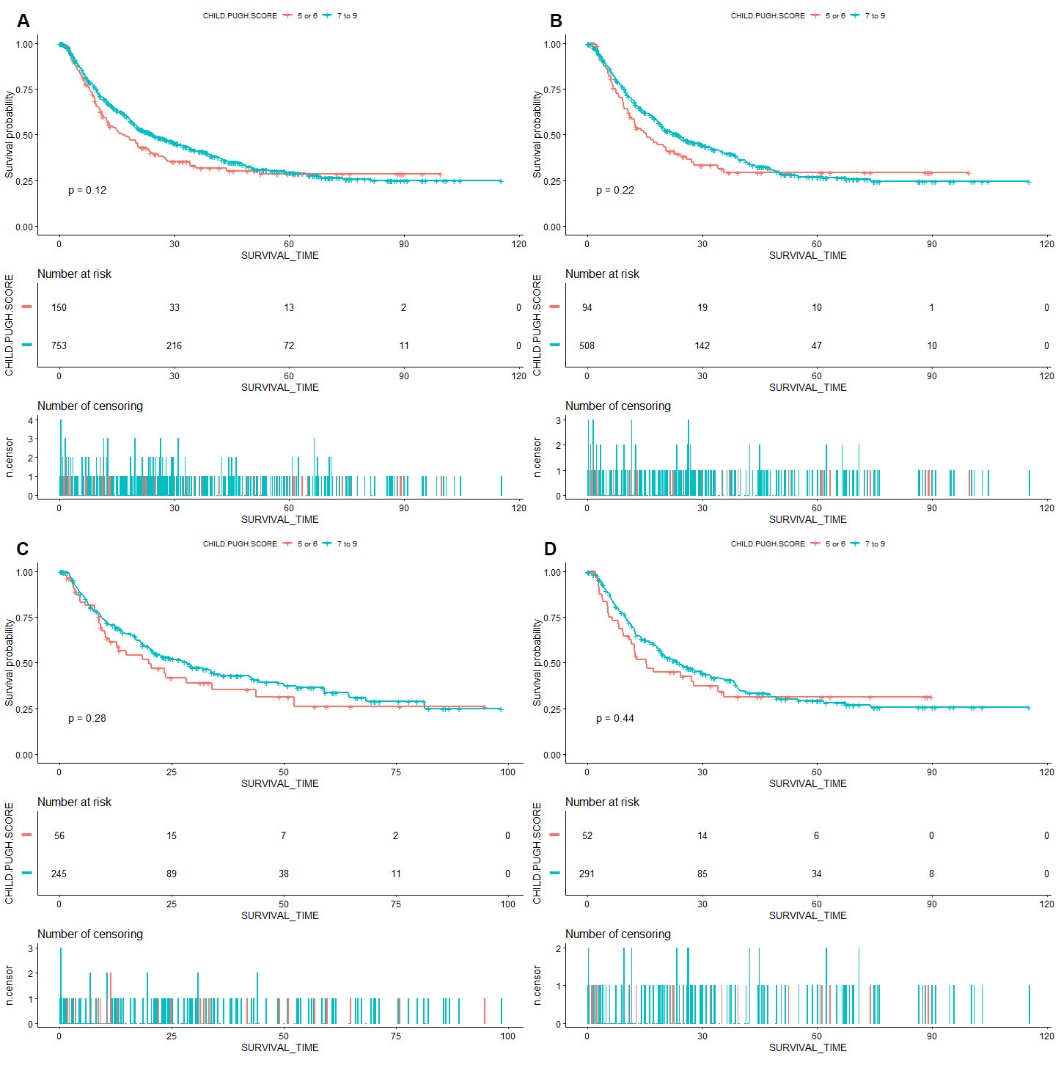

Supplement: Supplementary file 1 [file DataSheet_1.docx]
